# Carbolic Acid in Metrorrhagia and Menorrhagia

**Published:** 1872-11

**Authors:** C. S. Strother

**Affiliations:** Barnesville, Georgia


### CARBOLIC ACID IN METRORRHAGIA AND MENORRHAGIA.

BY C. S. STROTHER, M.D., BARNESVILLE, GEORGIA.

[Read Before the Middle Georgia Medical Society, May 15th, 1872.]

Under the Section of Materia Medica, I desire to call the attention of the Society to the therapeutic action of carbolic acid in metrorrhagia and menorrhagia; though the idea is not original with me, yet the prosecution of it has been pleasant to me, and no doubt the results will be gratifying to the father of the thought.

At the meeting of the Atlanta Academy of Medicine, July 28th, 1871, Professor J. G. Westmoreland, M.D., reported two cases of menorrhagia treated successfully with this agent, two grains being given three or four times daily, largely diluted in water. Since this report, I have neither heard nor read anything further on the subject.

During the greater part of 1871, I had, at various times, Miss ——, aged forty-three years, under treatment for menorrhagia. She was menstruating every two or three weeks, wtth the most excessive flow that I have ever seen attend the catamenial period, which would so prostrate her in twenty-four or forty-eight hours that she could not be raised up in bed without bringing on syncope; and it must be borne in mind that she was a large, muscular, active woman, when in health.

I gave her ferruginous tonics for months at a time, especially the tinctura ferri chloridi, and during the menstrual period, she took large doses of fluid extract ergot and acid sulph. aro., which was persisted in for days, very often with very little apparent benefit. Lead and opium were not given in combination, for she could not bear opium, or any of its preparations; it was also contra-indicated by her extreme and stubborn costiveness. I suspected polypus, and examined my patient with speculum, but found nothing to verify my apprehensions.

After reading Professor Westmoreland's report, referred to above, in the September number (1871) of the ATLANTA MEDICAL AND SURGICAL JOURNAL, and the treatment to which I had already subjected my patient had proven so unsatisfactory, I resolved to try the carbolic acid, and had prepared the following:

| | |
|---|---|
| ℞—Acid carbolic, | gtts. xx |
| Pure glycerin, | f℥j |

S.—Thirty to forty drops in water, four or five times daily.

I directed her to take this at her next period, should the flow become excessive. The flow came on profusely, as I expected, after a lapse of only two weeks; but imagine my gratification when she informed me that the first dose greatly diminished the flow, and the second and third checked it entirely. In the next few days, there was one faint effort at return, when one dose of the mixture checked it. The next return was three weeks off, which was easily controlled by the mixture. Her general health began to improve at once, menstruating regularly at intervals of not less than three nor more than four weeks—this happy result being accomplished after a twelve-month of almost unceasing drainage, prostration and suffering, with no other remedial agent than the one under consideration. I saw her six weeks since (April 1st), and she informed me that the tendency to menorrhagia had ceased entirely, and that she was in better health than for two years previous.

The next case treated by this remedy I had attended some months before, in her third confinement. She left her bed at the end of the third week, and began to do general domestic drudgery—such as cooking, making up beds, drawing water, etc.—which brought on a serious metrorrhagia, which she suffered from for a month or more, until she was very much prostrated, actually tottering with feebleness—her false modesty deterring her from seeking medical aid; but she finally reported her condition to me through the medium of a lady friend, when I prescribed the same mixture as for my first patient, requesting her to report to me very soon the action of the medicine. The message was, "the medicine had acted like a charm;" the second dose had checked the flow, the pain and weakness in her back disappearing immediately.

The third case was a colored woman, aged forty years; is the mother of six children; has had no children in fourteen years. She is large and stout, weighing one hundred and eighty pounds. For several years past she has been troubled with profuse menorrhagia, causing her to take her bed at each menstrual period, from extreme debility, the least exercise causing the flow to become alarming. I had treated her on several occasions with very indifferent success; so at the menstrual return, April 6th, 1872, I prescribed a similar mixture, of about the same proportions, as the one above. She was suffering very much with weakness, but had not yet taken her bed. The second dose relieved the pain and checked the flow considerably; and after using the medicine for twenty-four hours, the flow ceased to be of consequence.

Since writing the report of the above cases, I have treated a number of similar ones, the results in all of them tending to confirm the efficacy of the remedy. In some cases, the carbolic acid seemed to have failed at first; but after sometimes increasing the dose, or a longer persistence in the treatment, the results were about the same as those enumerated in the cases already reported.

